# *PfbHLH131* Mediates the Biosynthesis of Fragrance Compounds in *Primula forbesii* Franch

**DOI:** 10.3390/genes17070785

**Published:** 2026-07-08

**Authors:** Yu He, Wanqing Deng, Yuanzhi Luo, Benyue Ma, Zhuoxuan Li, Hongchen Yang, Yuanzhi Pan, Beibei Jiang, Pei Tu, Yin Jia

**Affiliations:** 1 Chengdu Botanical Garden (Chengdu Park City Botanical Science Research Institute), Chengdu 610083, China; heyu_henry@163.com; 2 College of Landscape Architecture, Sichuan Agricultural University, Chengdu 611130, China; dengwanqing29@163.com (W.D.); lyuanzhi2023@163.com (Y.L.); 18736533016@163.com (B.M.); lilizhuoxuan@163.com (Z.L.); 2022210011@stu.sicau.edu.cn (H.Y.); scpyzls@163.com (Y.P.); 13786@sicau.edu.cn (B.J.)

**Keywords:** *P. forbesii*, bHLH, floral fragrance, VIGS, gene expression regulation

## Abstract

**Objectives**: This study aims to provide a theoretical basis for a deeper understanding of the transcriptional regulatory network underlying the formation of *Primula forbesii* floral scent, and also offers important genetic resources for the molecular improvement of floral scent traits in *Primula* species. **Methods**: Using the *P. forbesii* cultivar ‘Pink violet’ as experimental material, we cloned *PfbHLH131* and analyzed its expression pattern. We also validated its function via virus-induced gene silencing (VIGS) and used gas chromatography–mass spectrometry (GC-MS) to analyze changes in floral aroma components in silenced plants. Additionally, we detected the expression levels of key structural genes involved in floral aroma biosynthesis in silenced plants via qRT-PCR to elucidate the regulatory role of *PfbHLH131* in the biosynthesis of *P. forbesii* floral aroma. **Results**: The cloned *PfbHLH131* open reading frame is 922 bp in length, encoding a total of 307 amino acids, and contains a bHLH_AtBPE_like domain characteristic of the bHLH gene family. Quantitative real-time PCR (qRT-PCR) revealed that *PfbHLH131* is highly expressed in floral organs, peaking during full bloom. Subcellular localization studies indicated that it is localized to the nucleus. VIGS-mediated transient silencing of *PfbHLH131* significantly reduced the release of terpenoid and phenylpropanoid floral odor compounds and suppressed the expression of multiple key structural genes in both synthetic pathways. **Conclusions**: *PfbHLH131* is a positive regulator of scent biosynthesis in *P. forbesii*.

## 1. Introduction

Floral fragrance is a complex mixture of various low-molecular-weight volatile organic compounds released by plants [1]. As a key secondary metabolite, it not only significantly enhances the aesthetic and commercial value of ornamental plants but also plays a central role in ecological interactions. Specifically, specific floral scents can precisely attract pollinators, ensuring the success of sexual reproduction and helping plants cope with biotic and abiotic stresses [2,3,4]. At the same time, these aromatic compounds serve as important raw materials for the perfume, cosmetics, and food and pharmaceutical industries, harboring immense economic potential [5]. Despite the significant value of floral scents, research on them has been relatively scarce compared to studies on flower color, shape, and flowering time, due to their complex and variable nature. Currently, approximately 1700 plant floral volatile compounds have been identified, which are primarily classified into three major categories based on their synthetic precursors and metabolic pathways: terpenes, phenylpropanoids, and fatty acid derivatives [2]. Terpenoid compounds are the most diverse group, and their synthesis relies on two distinct yet interconnected pathways within the cell. In the cytoplasm, the mevalonate pathway, starting from acetyl–CoA, ultimately synthesizes sesquiterpene precursors such as farnesyl pyrophosphate [6]. In the plastids, the methyl erythritol phosphate pathway utilizes pyruvate and glyceraldehyde-3-phosphate to generate precursors for monoterpenes and diterpenes, such as geranyl pyrophosphate [7]. The isopentenyl pyrophosphate and its isomers produced by these two pathways are ultimately catalyzed by terpenesynthases to form structurally diverse terpenoid volatiles [8,9]. Phenylpropanoid compounds constitute the second largest class of floral aroma components, and their synthesis originates from phenylalanine derived from the shikimate pathway. This pathway begins with a deamination reaction catalyzed by phenylalanine deaminase, yielding trans-cinnamic acid as a key intermediate. Subsequently, through a series of modifications by methyltransferases, reductases, and acyltransferases, it is converted into aromatic volatile compounds such as benzaldehyde and phenethyl alcohol [10,11,12]. Fatty acid derivatives are primarily synthesized from C18 fatty acids such as linoleic acid and linolenic acid. These serve as substrates in the lipoxygenase pathway to generate hydroperoxides, which are then catalyzed by a cascade of enzymes—including lyases, dehydrogenases, and acyltransferases—to form small-molecule volatiles, including C6/C9 aldehydes, alcohols, and esters [13]. Currently, the synthetic pathways for floral compounds have been largely elucidated, but the transcriptional regulators upstream of structural genes remain to be further identified.

Transcription factors (TFs) are essentially protein molecules located within the cell nucleus. Their primary function is to specifically bind to cis-acting elements in the promoter regions of target genes, ultimately regulating the spatiotemporal expression of those genes. Currently, several transcription factors have been shown to be involved in the biosynthesis of floral compounds, including MYB [14], bHLH [15], WRKY [16], ERF/AP2 [17], and bZIP [18]. Among the numerous transcription factor families, the basic helix-loop-helix (bHLH) family has attracted significant attention due to its conserved domains and extensive functions in growth and development, stress responses, and secondary metabolism [19,20,21,22,23,24,25]. However, research on the biosynthesis of floral aroma compounds within this family remains relatively scarce, having been conducted only in a limited number of ornamental plants. For example, in *Arabidopsis thaliana*, it was found that *AtMYC2* can directly bind to the promoters of the sesquiterpene synthase genes *AtTPS21* and *AtTPS11* and activate their expression, thereby inducing an increase in the release of sesquiterpenes [26]. To investigate the regulation of floral fragrance biosynthesis in *Phalaenopsis bellina*, researchers conducted comparative transcriptomic analyses of fragrant and non-fragrant *P. bellina* plants, identifying five highly expressed transcription factors: *PbbHLH4*, *PbbHLH6*, *PbbZIP4*, *PbERF1*, and *PbNAC1*. Through heterologous transient expression experiments, it was found that *PbbHLH4* can induce the production of monoterpenes in scentless orchids, increasing monoterpene production by 950-fold [27]. In a study of *Lilium* ‘Siberia’, researchers functionally characterized the two genes *LibHLH22* and *LibHLH63* and performed transient overexpression and virus-induced gene silencing (VIGS) of these genes. They found that these two genes significantly promote the expression of *LiDXR* and *LiTPS*, thereby enhancing the release of floral fragrance [28]. Similarly, in a study of *Dendrobium officinale*, it was found that *DobHLH4* is highly expressed in petals and can bind to the promoter to upregulate the expression of *DoTPS10*, thereby increasing linalool content and promoting the release of floral fragrance [29].

*Primula* is one of the world’s three most famous alpine flowers and derives its name from its early spring blooming. After years of introduction, domestication, and cultivation, a large number of *Primula* species are now available in the horticultural market. However, their ornamental traits are primarily focused on their vibrant and diverse flower colors, while fragrant characteristics are generally lacking. *P. forbesii* Franch. is a native plant of Sichuan and Yunnan provinces in China. Its intense fragrance makes it an ideal material for researching and improving the floral aroma of Primula species. In this study, we identified the candidate gene *PfbHLH131*, which is highly expressed specifically in floral organs, based on whole-genome and transcriptomic data. We analyzed the spatiotemporal expression patterns and subcellular localization of *PfbHLH131* and validated its function using virus-induced gene silencing (VIGS) technology. The aim is to elucidate the regulatory role of *PfbHLH131* in the synthesis of *P. forbesii*’s floral fragrance, thereby providing a basis for a deeper understanding of the transcriptional regulatory network underlying the formation of *P. forbesii*’s floral fragrance.

## 2. Materials and Methods

### 2.1. Plant Materials

*P. forbesii* ‘Pink violet’ was used as the experimental material. It was propagated by seed in the greenhouse of the College of Landscape Architecture at Sichuan Agricultural University (30°42′ N, 103°51′ E) using a growing medium of peat moss and perlite (mixed in a 2:1 ratio). The temperature was maintained at 18 ± 2 °C, with a relative humidity of 80%, and standard water and fertilizer management practices were followed. Flower buds, early-flowering, peak-flowering, and late-flowering stages were selected as floral organs representing different developmental phases, while roots, flower stalks, leaves, and flowers were used as organ samples. These were rapidly frozen in liquid nitrogen and stored at −80 °C for RNA extraction and spatiotemporal expression pattern analysis. *Nicotiana benthamiana* plants, used for subcellular localization studies, were grown in a greenhouse under environmental conditions of 25 ± 2 °C and a 12/12 h photoperiod; they were used for injection experiments four weeks after planting.

### 2.2. Cloning and Bioinformatics Analysis of PfbHLH131

Total RNA was extracted from *P. forbesii* flowers using the TIANGE Centrifugal Column Type S Polysaccharide and Polyphenol RNA Extraction Kit (Tiangen Biotech, Beijing, China). RNA concentration was measured using a Nanodrop 2000 ultra-micro spectrophotometer (Thermo Fisher Scientific, Waltham, MA, USA), and RNA samples with a concentration of 600 or higher, with an OD260/280 ratio between 1.8 and 2.1 and an OD260/230 ratio between 1.9 and 2.1. The extracted RNA was reverse-transcribed into cDNA using the Evo M-ML V Reverse Transcription Master Mix Kit (Accurate Biology, Changsha, China), followed by PCR amplification and cloning according to the following protocol: 94 °C for 3 min; 94 °C for 30 s; annealing at 60 °C for 30 s; 72 °C for 1 min, repeated 35 times; 72 °C for 10 min; cool to 4 °C. The PCR products were purified and ligated into the pTOPO-TA/Blunt cloning vector (Aidlab Biotech, Beijing, China); the primer sequences used are shown in Supplementary Table S1.

The conserved domains, physicochemical properties, transmembrane domains, signal peptide, phosphorylation sites, secondary structure characterization, and tertiary structure modeling of *PfbHLH131* were determined using the CD-Search tool (NCBI, Bethesda, MD, USA), Expasy ProtParam tool (NCBI, Bethesda, MD, USA), TMHMM v.2.0 (DTU, Copenhagen, Denmark), SignalP 6.0 (DTU, Copenhagen, Denmark), NetPhos 3.1 Server (DTU, Copenhagen, Denmark), SOPMA (IBCP, CNRS, Lyon, France), and SWISS-MODEL (SIB, Geneva, Switzerland) from the NCBI database, respectively. The sequence of the *PfbHLH131* homolog was downloaded from GenBank, and amino acid sequence alignment was performed using DNAMAN (Version 8.0). The sequences of the *A. thaliana bHLH* family proteins were downloaded from PlantTFDB 5.0, and a phylogenetic tree was constructed using MEGA (Version 11). See Table S2 for the corresponding accession numbers or web links.

### 2.3. Analysis of the Spatiotemporal Expression Pattern of PfbHLH131

qRT-PCR analysis was performed using *PfEIF5A* as an internal control [30]; the primer sequences used are shown in Supplementary Table S1. qRT-PCR assays were conducted using the SYBR Green Pro Taq HS qPCR Master Mix (ACCURATE, Changsha, China) on a real-time quantitative PCR instrument (CFX Connect Bio-Rad, Hercules, CA, USA). The reaction program was as follows: 94 °C for 30 s; 94 °C for 5 s, 60 °C for 30 s, for 45 cycles. The assay was performed in triplicate, and relative gene expression levels were calculated using the 2-ΔΔCt method [31]. In addition, floral organs and S1 samples were used as calibration samples.

### 2.4. Subcellular Localization Analysis of PfbHLH131

The *PfbHLH131* gene was cloned into the KpnI/SalI sites of the pCAMBIA2300-GFP vector using a seamless cloning method, and the fusion plasmid was subsequently transformed into *Agrobacterium* strain GV3101. The primers used to construct the subcellular localization vector are listed in Supplementary Table S1. The Agrobacterium suspension was cultured overnight, resuspended in a solution containing 10 mmol/L MES, 10 mmol/L MgCl_2_, and 200 μmol/L AS to an OD600 of 1.0, and allowed to stand at room temperature for 1–2 h. It was then used to inoculate 4-week-old *N. benthamiana* plants. After inoculation, the plants were cultured in the dark for 24 h, followed by 1–2 days of light-dependent growth. Finally, GFP fluorescence signals were observed using a confocal microscope (Leica TCS SP8, Wetzlar, Germany).

### 2.5. VIGS-Mediated Transient Silencing of PfbHLH131

The target fragment of *PfbHLH131* was inserted into the EcoRI/XhoI sites of the pTRV2 vector to construct the pTRV2-*PfbHLH131* recombinant plasmid. Cultivate Agrobacterium suspensions containing pTRV1, pTRV2, and pTRV2-*PfbHLH131* separately until OD600 = 1.0, then resuspend them in infection medium (10 mmol/L MES, 10 mmol/L MgCl_2_, and 200 μmol/L AS). Mix pTRV2-*PfbHLH131*: pTRV2 was mixed with pTRV1 in a 1:1 ratio. When the flower stalks of *P. forbesii* had emerged but the flowers had not yet opened, the mixed bacterial suspension was injected into the plants via the underside of the leaves using a syringe. After inoculation, the plants were kept in the dark for 24 h and then transferred to a controlled environmental chamber for normal cultivation. After 30–40 days, in plants injected with pTRV1 + pTRV2-PfPDS, PfPDS gene silencing caused characteristic bleaching of newly emerged leaves, confirming that the virus successfully infected this species and achieved systemic gene silencing, indicating that the silencing system is functioning effectively. PCR-positive identification was performed using TRV-specific primers (Supplementary Table S1). Petals were collected from positive plants at peak flowering, RNA was extracted and reverse-transcribed, and the silencing efficiency of the *PfbHLH131* gene in positive plants was detected via qRT-PCR. Approximately 0.3 g of fresh petal samples were weighed and placed in 20 mL headspace vials, to which 5 μL of 0.5% ethyl decanoate was added. Headspace solid-phase microextraction coupled with gas chromatography–mass spectrometry (GC-MS) was employed to analyze floral aroma components. Compounds were identified using the NIST spectral library, and the relative content of each component (μg/g FW) was calculated using the internal standard method. The calculation formula is as follows: Relative content of each aromatic compound = (peak area of each compound/peak area of the internal standard) × (concentration of the internal standard × volume of the internal standard/mass of the sample). Concurrently, qRT-PCR was used to detect the expression levels of key structural genes involved in floral aroma synthesis in the petals of silenced plants and control plants (Supplementary Table S1).

## 3. Results

### 3.1. Bioinformatic Characterization of PfbHLH131

**Figure 1:**
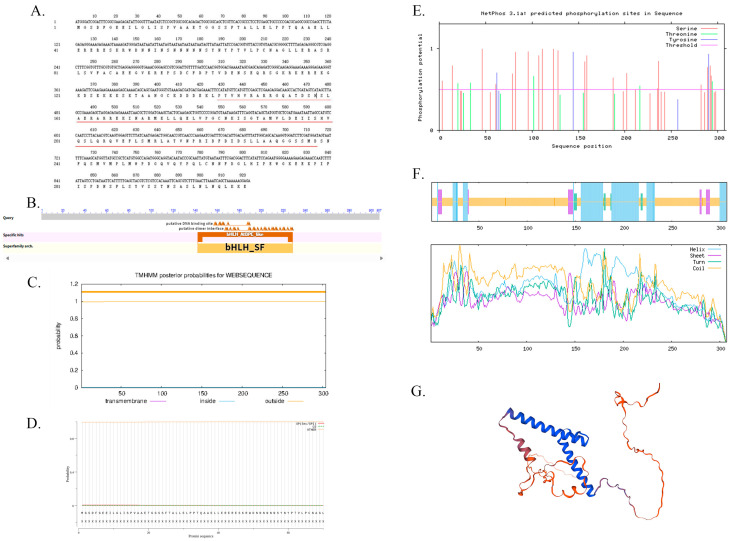
*PfbHLH131* protein characterization. (**A**) Sequence characteristics of *PfbHLH131* (The red underlined parts are the conserved domain amino acid sequences); (**B**) Conserved domains analysis of *PfbHLH131* protein; (**C**) Predicted transmembrane structure of the *PfbHLH131* protein; (**D**) Signal peptide prediction for *PfbHLH131*; (**E**) Prediction of phosphorylation sites in *PfbHLH131*; (**F**) Secondary structure prediction; (**G**) 3D model building.

Based on whole-genome data and four sets of flowering-stage transcriptomic data from *P. forbesii*, this study identified a bHLH transcription factor whose expression pattern during full bloom aligns with the synthesis and release of floral odor compounds in *P. forbesii*. Based on its chromosomal localization, it was named *PfbHLH131*. The open reading frame of *PfbHLH131* is 922 bp and encodes 307 amino acids (Figure 1A). Analysis of conserved domains indicates that it belongs to the bHLH superfamily and possesses a typical BPE domain (Figure 1B). The theoretical isoelectric point (pl) of *PfbHLH131* is 5.60, its theoretical relative molecular weight is 34.21 kDa, the total number of positively charged residues (Arg + Lys) is 39, and the total number of negatively charged residues (Asp + Glu) is 44. Furthermore, *PfbHLH131* has an instability index of 73.09 (>40) and a total average hydrophilicity of −0.665 (<0), indicating that it is an unstable hydrophilic protein. The transmembrane structure of the protein indicates that *PfbHLH131* lacks transmembrane helices, does not undergo transmembrane movement, is not a membrane protein, and lacks a distinct signal peptide sequence, classifying it as a non-secretory protein (Figure 1C,D). The phosphorylation site prediction results indicate the presence of 29 phosphorylation sites (Figure 1E). The protein secondary structure reveals 83 α-helices, 8 β-turns, 16 extended chains, and 203 random coils (Figure 1F), as well as the predicted tertiary structure shown in Figure 1G.

**Figure 2:**
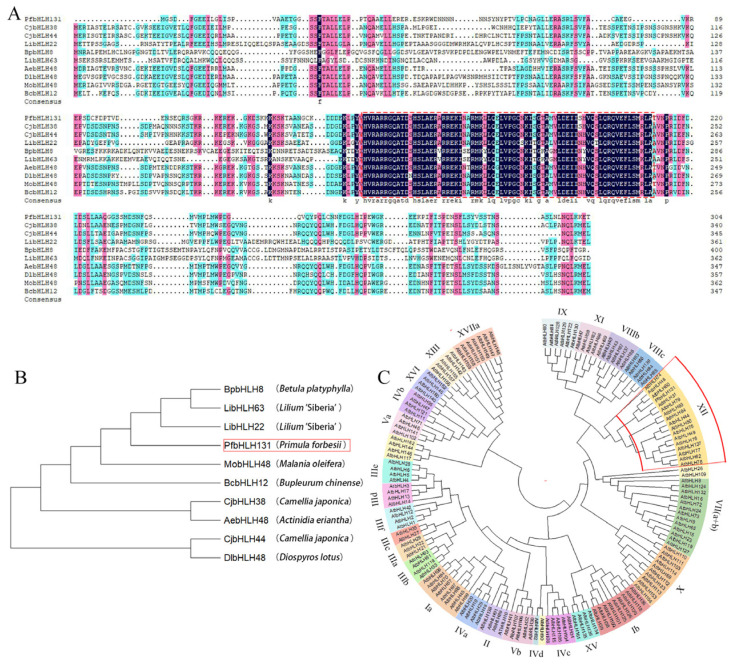
Gene sequence analysis. (**A**) *PfbHLH131* homologous protein sequence alignment, the red box indicates the BPE domain sequence; (**B**) Phylogenetic tree of the *PfbHLH131* protein; (**C**) Comparison of the *PfbHLH131* protein with bHLH family proteins of *A. thaliana*. The red box indicates that these genes belong to the bHLH subfamily XII.

Phylogenetic tree analysis indicates that *PfbHLH131* belongs to subfamily XII (Figure 2C). Multiple sequence alignment reveals that *PfbHLH131* is closely related to Lilium (*Lilium*) *LibHLH22* and *LibHLH63* (Figure 2B), as well as *Betula platyphylla BpbHLH8*, and all possess the BPE domain common to bHLH family members (Figure 2A).

### 3.2. Analysis of the Spatiotemporal Expression Pattern and Subcellular Localization of PfbHLH131

**Figure 3:**
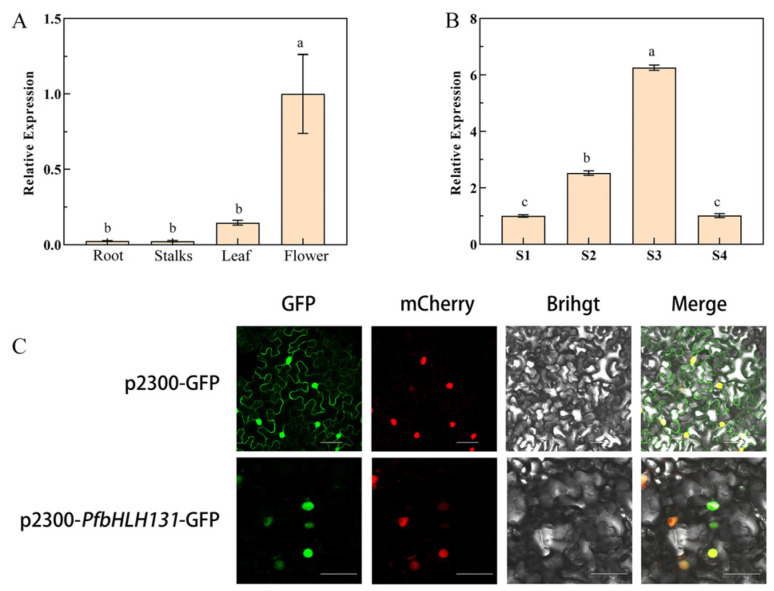
Spatiotemporal expression and subcellular localization of *PfbHLH131*. (**A**) Expression of *PfbHLH131* in different organs of *P. forbesii.* The letters in the figure represent the salience of the components; (**B**) Expression of *PfbHLH131* at different flowering stages of *P. forbesii* (S1, petals of the bud stage; S2, first flowering stage; S3, full blooming stage; S4, late flowering stage). The different lowercase letters in the figure indicate significant differences at the *p* < 0.05 threshold; (**C**) Subcellular localization of *PfbHLH131*. “GFP” stands for green fluorescent protein, which indicates the distribution of this protein within the cell. “mCherry” indicates the location of the cell nucleus within the cell. “Bright” refers to a bright-field image that shows the morphological structure of the cells. “Merge” is a composite image created by overlaying the GFP, mCherry, and Bright images. Scale bar = 50 µm.

qRT-PCR analysis indicated that *PfbHLH131* is expressed in the roots, flower stalks, leaves, and floral organs of *P. forbesii*, but exhibits significant organ specificity. The relative expression level of this gene was highest in floral organs, significantly higher than in the three other organs—flower stalks, leaves, and roots—among which there were no significant differences in relative expression levels (Figure 3A).

The development of a flower is divided into four stages: S1–S4 represent the bud stage, early flowering stage, full blooming stage, and late flowering stage, respectively. In stage S1, the petals are enclosed by the sepals, and neither the sepals nor the petals have expanded. In stage S2, the sepals spread out, the petals elongate, and the flower is half-open. At stage S3, the sepals and petals are fully expanded, and the petals are in full bloom. At stage S4, the petals contract, change color, curl outward, wither, and are prone to falling off. The expression level of *PfbHLH131* was significantly higher in the S3 period than in the S2 period, and significantly higher in the S2 period than in both the S1 and S4 periods; there was no significant difference between the S1 and S4 periods. Overall, the expression level of *PfbHLH131* undergoes dynamic changes, gradually increasing from the bud stage to full bloom, peaking at full bloom, and subsequently declining during the late flowering stage (Figure 3B).

Confocal scanning microscopy was used to detect GFP fluorescence signals in transiently transformed *N*. *benthamiana* plants. The results showed that the *PfbHLH131* fusion protein was primarily localized to the cell nucleus (Figure 3C).

### 3.3. Functional Validation of PfbHLH131 Silencing via VIGS

**Figure 4:**
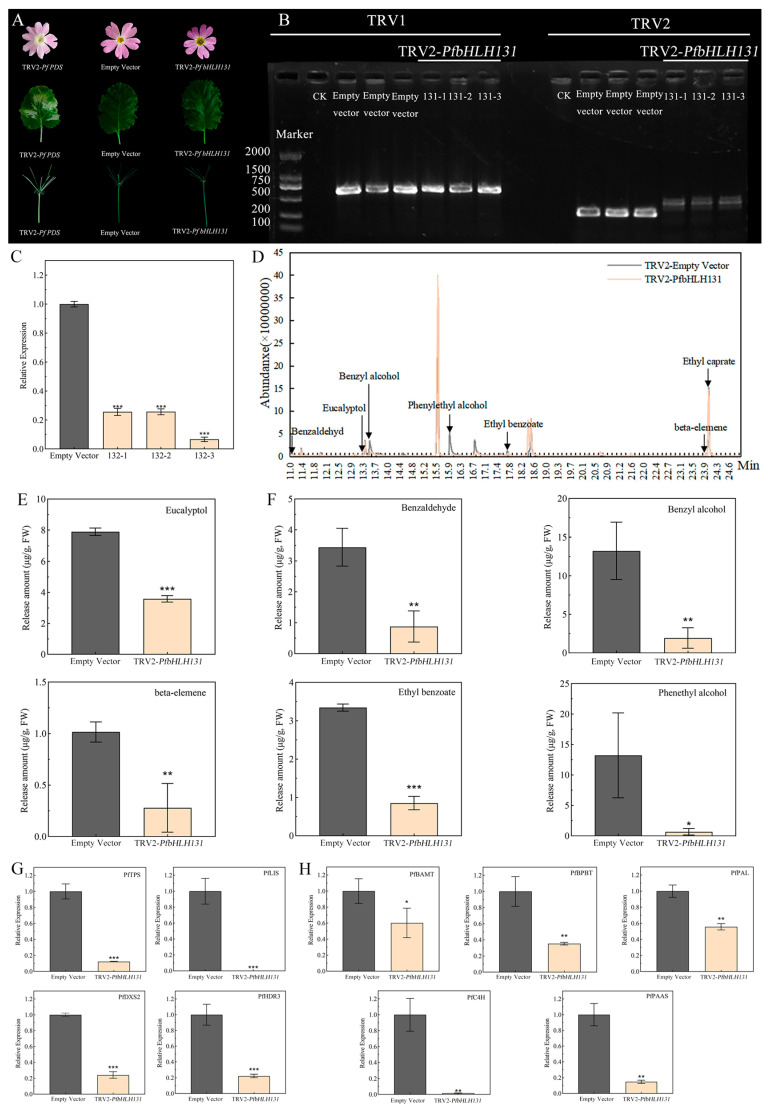
Transient silencing of *PfbHLH131* in *P. forbesii*. (**A**) Phenotypes of the whole plant, flowers, stems, and leaves after the transient silencing of *PfbHLH131* in *P. forbesii*; (**B**) Detection of PCR-positive results in the *PfbHLH131* knockdown line. Normal plants that were not injected with the staining solution were used as the control (CK); (**C**) Detection of the relative expression of *PfbHLH131* in different lines; (**D**) The floral contents of *PfbHLH131*-silenced plants were detected by GC-MS; (**E**) Relative contents of key terpenoids in flowers of control and silenced plants; (**F**) Relative content of key phenylpropanoid compounds in flowers of the control group and plants subjected to silencing treatment; (**G**) Expression of key genes involved in the biosynthesis of terpenoids; (**H**) Expression of key genes involved in the biosynthesis of propylbenzene compounds. Data are expressed as means ± standard deviations of three replicates (***, *p* < 0.001; **, *p* < 0.01; *, *p* < 0.05).

Thirty days after injection of the infection solution, plants injected with pTRV2-*PfPDS* exhibited typical leaf and flower stalk bleaching, confirming the effectiveness of the VIGS system, whereas plants injected with pTRV2-*PfbHLH131* showed no obvious morphological differences compared to the empty vector control (Figure 4A). The presence of the viral vector in the silenced plants was confirmed by PCR (Figure 4B). qRT-PCR analysis showed that, compared to the empty vector control, the expression levels of the target gene in the three independent *PfbHLH131* silenced lines decreased by 74%, 74%, and 93%, indicating that the *PfbHLH131* gene was effectively silenced (Figure 4C).

GC-MS analysis indicated that *PfbHLH131* silencing significantly reduced the release of major floral aroma compounds from *P. forbesii* petals (Figure 4D). Among terpenes, the release of eucalyptol and β-elemene decreased by 40.68% and 72.49%, respectively (Figure 4E). Among the phenylpropanoids, phenethyl alcohol showed the greatest decrease, followed by phenylmethanol, ethyl benzoate, and benzaldehyde, with reductions of 94.84%, 85.34%, 74.5%, and 74.4%, respectively (Figure 4F).

To investigate the molecular mechanism by which *PfbHLH131* regulates floral fragrance biosynthesis, we examined changes in the expression of key genes in the relevant biosynthetic pathways. In *PfbHLH131* silenced plants, the expression of key genes in the terpenoid biosynthesis pathways was generally downregulated. Among them, the expression levels of the monoterpene synthase genes *PfLIS* and *PfTPS* decreased to 0.00-fold and 0.12-fold of the control, respectively. The key genes of the MEP pathway, *PfDXS2* and *PfHDR3*, also decreased to 0.24-fold and 0.22-fold of the control, respectively (Figure 4G). Concurrently, the expression of multiple genes in the phenylpropanoid synthesis pathway was also significantly suppressed, with the relative expression levels of *PfBAMT*, *PfPAAS*, *PfC4H*, *PfBPBT*, and *PfPAL* decreasing to 0.61, 0.12, 0.04, 0.56, and 0.63 times that of the control, respectively (Figure 4H).

## 4. Discussion

As a defining characteristic of *P. forbesii*, floral fragrance not only plays a crucial role in regulating pollination and enhancing environmental adaptability, but also serves as a key indicator for assessing their ornamental value, while simultaneously influencing their market potential and prospects for development and utilization. To date, there has been relatively little research on the floral aroma of *P. forbesii*. However, existing studies have preliminarily revealed the key regulatory network governing terpenoid metabolism in its flowers: based on transcriptomic sequencing, the key enzyme gene *PfDXS2* in the terpenoid biosynthetic pathway was successfully identified, and virus-induced gene silencing (VIGS) experiments confirmed that downregulation of its expression significantly reduces the relative content of the major monoterpenes linalool and α-pinene [32]. At the same time, the ethylene-responsive factor *PfERF106* was cloned and characterized for the first time from *P. forbesii*. Its expression is induced by exogenous ethylene, and bidirectional validation via transient silencing and heterologous overexpression demonstrated that it positively regulates the biosynthesis of various terpenoids, including linalool, linalyl acetate, and elemol [33]. The above study has laid the molecular foundation for an in-depth analysis of the transcriptional regulatory network governing terpenoid metabolism in *P. forbesii*. However, to date, there have been no reports on whether bHLH transcription factors are involved in the regulation of terpenoid metabolism in *P. forbesii*.

bHLH is one of the largest families of transcription factors; it is widely distributed in eukaryotes and plays a key role in plant growth, development, and secondary metabolism [34,35,36]. Two functional domains are conserved in bHLH transcription factors: the basic domain, which mediates sequence-specific DNA binding, and the HLH domain, which is involved in homodimerization or heterodimerization [37]. *PfbHLH131*, identified in this study, belongs to bHLH subfamily XII and shares the BPE domain with other members of the bHLH family. Multiple sequence alignment revealed that *PfbHLH131* is closely related to two *Lilium* genes, *LibHLH22* and *LibHLH63*, which are involved in the biosynthesis of terpenoid floral compounds [28]. Therefore, it is highly likely that *PfbHLH131* is involved in the biosynthesis of floral compounds in *P. forbesii*.

We further analyzed the spatiotemporal expression patterns and subcellular localization of *PfbHLH131* in different organs and at different stages of flower development. The results showed that *PfbHLH131* is expressed in four organs: roots, flower stalks, leaves, and flowers. Expression levels were highest in floral organs, significantly higher than in other organs, indicating a clear organ-specific expression preference, which suggests that it may be specifically involved in regulatory processes related to floral development. For example, in *Rhododendron* × *pulchrum* Sweet, multiple *RpbHLHs* genes exhibit significant spatiotemporal specificity in floral organs, and their expression levels are highly correlated with the dynamics of anthocyanin accumulation [38]. Similarly, in *Chimonanthus praecox*, multiple *CpbHLHs* genes exhibit differential expression patterns across different organs and stages of floral development, with some members confirmed to participate in the coordinated regulation of secondary metabolites such as flower color and fragrance [39]. In *Osmanthus fragrans*, *OfbHLH79* is most highly expressed in the petals; its overexpression significantly upregulates the expression of *OfXTH28*, which may be involved in regulating petal cell expansion during the flower opening process [40]. Relative expression levels across the four flowering stages show a trend of first increasing and then decreasing, peaking during full bloom. This pattern aligns with the fragrance release dynamics of *P. forbesii* and is highly similar to the expression characteristics of genes known to function in fragrance synthesis across multiple species. Such as *LibHLH22* and *LibHLH63* in *Lilium* [28], *D. officinale*’s *DobHLH4* [29], and *Petunia* × *atkinsiana*’s *PhbHLH19* [41], all of which are highly expressed in petals and promote the synthesis and accumulation of terpenoids and phenylpropanoids. Therefore, *PfbHLH131* may be involved in the biosynthesis of terpenoid and phenylpropanoid floral odorants in *P. forbesii*.

Transcription factors, as key molecules in the regulation of gene expression, exert their effects by binding to specific DNA sequences, and their subcellular localization directly determines how they function [42]. Subcellular localization results indicate that *PfbHLH131* is localized to the nucleus, consistent with the characteristic of transcription factors acting within the nucleus, and aligns with the nuclear localization of bHLH transcription factors in the vast majority of plants. For example, bHLH transcription factors in plants such as *Prunus avium* [43], *Camellia sinensis* [44], *Chrysanthemum morifolium* [45,46] and *Vitis vinifera* [47] are all localized to the nucleus. The nucleus is the primary site for gene transcription regulation. The nuclear localization of *PfbHLH131* indicates that it may possess transcriptional factor activity, enabling it to bind to DNA and regulate the expression of downstream genes, thereby influencing plant physiological processes. Combined with the consistency between its organ-specific expression pattern and the rhythm of floral fragrance release, the nuclear localization results further support the hypothesis that *PfbHLH131* may participate in the transcriptional regulatory network governing the formation of *P. forbesii* floral fragrance by directly regulating the expression of structural genes in the floral fragrance synthesis pathway.

In this study, we investigated the regulatory role of *PfbHLH131* in the biosynthesis of floral compounds in *P. forbesii* using virus-mediated gene silencing. The results showed that following silencing of the *PfbHLH131* gene, the release levels of both phenylpropanoid and terpenoid floral compounds in *P. forbesii* exhibited a significant downward trend, while the expression levels of several key structural genes in the floral compound biosynthetic pathways were also significantly downregulated. These findings provide new experimental evidence for the involvement of bHLH transcription factors in regulating plant floral aroma metabolism and lay the foundation for a deeper understanding of the molecular regulatory network underlying floral aroma formation in *P. forbesii*. bHLH transcription factors are a class of important transcriptional regulators in eukaryotes, widely involved in various biological processes such as plant growth and development, hormone responses, and the regulation of secondary metabolism. In recent years, the role of bHLH family members in floral fragrance regulation has gradually garnered attention. For example, in *C. praecox*, overexpression of *CpMYC2* significantly increased the content of linalool [48]. In *Lavandula angustifolia*, overexpression of *LaMYC4* significantly affected the production of volatile terpenes [49]. And in *Artemisia annua*, transient expression of *AabHLH1* significantly increased the expression of artemisinin [50]. Similarly, in *PfbHLH131*-silenced lines, β-elemene and eucalyptol levels decreased significantly, and *PfbHLH131* regulates a broad range of terpenoid biosynthesis genes, affecting not only downstream terpene synthase genes but also key enzyme genes in the upstream MEP and MVA pathways. The suggesting that these transcription factors may occupy upstream nodes in the terpenoid metabolic regulatory network, influencing terpenoid synthesis flux through multi-level regulation.

Current research has primarily focused on the regulation of terpenoid floral compounds by bHLH transcription factors, and reports on whether bHLH members participate in the metabolism of phenylpropanoid compounds are relatively limited. A recent study reported that *PhbHLH19* in Petunia may participate in the regulation of phenylpropanoid compound synthesis by activating *PhPAL2* expression [41], providing an important reference framework for this study. Silencing the *PfbHLH131* gene in *P. forbesii* not only resulted in a significant decrease in terpenoid content but also caused a marked reduction in the release of phenylpropanoid compounds, suggesting that *PfbHLH131* may simultaneously participate in the regulatory networks of multiple floral aroma metabolic pathways. In the *PfbHLH131*-silenced line, the contents of phenethyl alcohol and phenylmethanol both showed a significant decrease, and several key genes in phenylpropanoid metabolism, including *PfPAL*, *PfC4H*, *PfPAAS*, and *PfBPBT*, were significantly downregulated. This result is similar to the pattern of *ODO1*, *EOBI*, and *EOBII* regulating phenylpropanoid biosynthesis in Petunias, where *ODO1* activates the expression of multiple structural genes in the phenylpropanoid metabolic pathway [51]. Therefore, *PfbHLH131* may participate in the transcriptional regulation of the phenylpropanoid metabolic pathway in a similar manner, but its specific target genes and regulatory mechanisms require further verification.

## 5. Conclusions

In this study, we cloned and characterized the bHLH transcription factor *PfbHLH131* from *P. forbesii*. Spatiotemporal expression and subcellular localization analyses revealed that this gene is highly expressed in floral organs, reaching peak expression during full bloom, and is localized to the nucleus. Transient silencing of *PfbHLH131* via virus-induced gene silencing (VIGS) significantly reduced the release of terpenoid and phenylpropanoid floral odor compounds and suppressed the expression of multiple key structural genes in both synthetic pathways, indicating that *PfbHLH131* acts as a positive regulator of floral odor synthesis in *P. forbesii*. This study provides genetic resources for the molecular improvement of floral aroma traits in *Primula* species. Further research can explore the target genes directly regulated by this gene to elucidate its fine-tuning regulatory network.

## Data Availability

The original contributions presented in this study are included in the article/Supplementary Materials. Further inquiries can be directed to the corresponding authors.

## Supplementary Materials

The following supporting information can be downloaded at: https://www.mdpi.com/article/10.3390/genes17070785/s1, Table S1: Primers used in this study. Table S2: Gene name and gene accession number or web link.
