# Supplementary material for: PfbHLH131 Mediates the Biosynthesis of Fragrance Compounds in Primula forbesii Franch
Source: Genes (Basel). 2026 Jul 8;17(7):785. doi: 10.3390/genes17070785 (PMC13409557; doi:10.3390/genes17070785)
Supplement: Supplementary file 1 [file genes-17-00785-s001.zip › genes-4399364-supplementary.pdf]

Supplement Table S1 Primers used in this study

Function	Primer name	Sequence
Gene cloning	*PfbHLH131*-F	ATGGGATCGGATTTCGGCGA
	PfbHLH131-R	TCTCCTTTTTTAGCTGATTTAAGTTCAAAGAC
Spatio-temporal representation models qRT-PCR	*PfbHLH131*-F	CAACCGTCAACCCAAGAAT
	PfbHLH131-R	ACATGAGCGGCATAACCAT
pCAMBIA2300-GFP-*PfbHLH131*	*PfbHLH131*-F	ATGGGATCGGATTTCGGCGAA
	PfbHLH131-R	TCCTTTTTTAGCTGATTTAAGTTCAAAGACGC
PTRV2-*PfbHLH131*	*PfbHLH131*-F	GCTTCTAGAGAGGGAAAGAGAAAGTAAAAGATGG
	PfbHLH131-R	CCGATCTCTGCTCGCTATTTTCGT
PCR-positive result	TRV1-F	TTACAGGTTATTTGGGCTAG
	TRV1-R	CCGGGTTCAATTCCTTATC
	TRV2-F	TGTTTGAGGGAAAAGTAGAGAACGT
	TRV2-R	TTACCGATCAATCAAGATCAGTCGA
Terpene structural genes qRT-PCR	*PfLIS*-F	TAAGGCTCTTCATCAGGT
	PfLIS-R	CTCTTCAGCTTTAGGCAC
	PfTPS-F	CTACGGATACGAAAGGAA
	PfTPS-R	CTAATGCGTGAGCAACTA
	PfDXS2-F	GACGAAGAGCCCAACACT
	PfDXS2-R	AAGCATCGGTCAGGAAAC
	PfHHDR3-F	TCACAACCCAACAGTCA
	PfHHDR3-R	GATACCCAAGGGCAAG
Structural motifs of propylene-based compounds qRT-PCR	*PfPAL*-F	CCGAGCAACATAACCAAG
	PfPAL-R	AATGCCACCAAATAAGTAGA
	PfPAAS-F	TGGGTATCTTCTCCTTCA
	PfPAAS-R	ACTATCTGCTCCGTGTTT
	PfSAMT-F	AAATGGTTGCTCAGGGTA
	PfSAMT-R	CTCGAATAGTTTGGGTCA
	PfBPBT-F	TCTCCGACATCGACGACCAA
	PfBPBT-R	CTTCCCTCAGCCTACCAGCA
	PfC4H-F	AATGTTCCCATCACCC
	PfC4H-R	GCCCGAAGATTCTGTA

Supplement Table S2 Gene name and gene accession number or web link

Gene name	Accession number or Web link
BpbHLH8	MH113336.1
MobHLH48	XM_058126475.1
BcbHLH12	OQ718235.1
CjbHLH38	PP033389.1
AebHLH48	XM_057640791.1
CjbHLH44	PP033395.1
DlbHLH48	XM_052317516.1
LibHLH22 and *LibHLH63*	https://www.arabidopsis.org/browse/gene_family/bHLH
AtbHLHs	https://doi.org/10.3390/horticulturae9040459
